# Left Ventricular Remodeling Shortly after Open Mitral Valve Replacement for Rheumatic Mitral Stenosis

**DOI:** 10.21470/1678-9741-2020-0641

**Published:** 2021

**Authors:** Marcus Vinicius Silva Ferreira, Cláudio Ribeiro da Cunha, Gabrielle Santos Oliveira, Maria Estefânia Otto, Fernando Antibas Atik

**Affiliations:** 1 Department of Cardiovascular Surgery, Instituto de Cardiologia do Distrito Federal, Brasília, DF, Brazil.; 2 Department of Echocardiography, Instituto de Cardiologia do Distrito Federal, Brasília, DF, Brazil.

**Keywords:** Mitral Valve Stenosis, Stroke Volumes, Mitral Valve, Odds Ratio, Rheumatic Diseases

## Abstract

**Introduction:**

Left ventricular dysfunction after surgical treatment of mitral stenosis is uncommon. We intend to determine the pattern of left ventricular remodeling, shortly after open mitral valve replacement for rheumatic mitral stenosis, with in-hospital postoperative outcomes and the determinants of postoperative worsening of left ventricular ejection fraction.

**Methods:**

From January 2008 to January 2015, 107 adult patients with rheumatic mitral stenosis were submitted to open mitral valve replacement. Their mean age was 45±11 years and 93 (86.9%) were women. Left ventricular morphology and function were studied longitudinally with echocardiography. The end point was postoperative worsening of left ventricular ejection fraction, defined by a decrease of 10% compared to preoperative basal assessment. Determinants of worsening left ventricular ejection fraction were determined by multivariable logistic regression analysis.

**Results:**

The end point occurred in 18 patients (16.8%). We tested clinical and echocardiographic parameters to verify independent variables related to the decrease in postoperative ejection fraction. Lower body weight (*P*=0.005; odds ratio [OR]=0.89) and smaller preoperative mitral valve area (*P*=0.02; OR=0.02) were independent predictors of left ventricular dysfunction. These patients presented higher mortality and morbidity rates.

**Conclusion:**

Left ventricular remodeling patterns differed among patients with predominant rheumatic mitral stenosis undergoing open mitral valve replacement. Lower preoperative body weight and mitral valve area were independent determinants of deteriorating ejection fraction with increased end-systolic volumes, indicating that this specific problem may occur in anthropometric smaller patients with more extensive rheumatic disease.

**Table:** 

Abbreviations, acronyms & symbols				
CI	= Confidence interval		MR	= Mitral regurgitation
EF	= Ejection fraction		MS	= Mitral stenosis
ICU	= Intensive care unit		MVA	= Mitral valve area
LA	= Left atrial		NYHA	= New York Heart Association
LAV	= Left atrial volume		OR	= Odds ratio
LV	= Left ventricular		PASP	= Pulmonary artery systolic pressure
LVEDD	= Left ventricular end-diastolic diameter		PHT	= Pressure half-time
LVEDV	= Left ventricular end-diastolic volume		TR	= Tricuspid regurgitation
LVEF	= Left ventricular ejection fraction		VTILVOT	= Velocity-time integral for left ventricle outflow tract
LVESD	= Left ventricular end-systolic diameter		VTIPrMV	= Velocity-time integral for mitral valve prosthesis
LVESV	= Left ventricular end-systolic volume			

## INTRODUCTION

The hallmark morphology of rheumatic mitral stenosis (MS) results from chronic endocardial insult in rheumatic fever carditis; commissural fusion, thickening, and narrowing of the valve leaflets lead to obstruction of left ventricular (LV) filling^(1,2)^. As opposed to mitral regurgitation, in which LV dysfunction may occur after surgery^(3)^ (as a compensatory mechanism of reduced end-diastolic volume), MS does not hold the same pathophysiological principle.

In MS, reduced stroke volume usually relates to reduced LV preload, rather than ventricular contractile impairment. However, some patients might present true systolic dysfunction that is independent from LV preload^(2)^. The exact mechanism remains unclear, but it is probably multifactorial. Earlier studies found that these patients exhibit larger end-systolic volumes and lower ejection fractions (EF) with rigidity of the LV posterobasal wall at ventriculography^(4)^; a diastolic dysfunction marked by altered ventricular compliance and elevated end-diastolic pressure^(5)^, with preserved EF and normal end-systolic volume^(6)^. Heart failure with reduced or preserved EF in MS has been linked to chronic LV underfilling, endomyocardial fibrosis, subvalvular apparatus rigidity, and elevated right ventricular pressure^(4,6-8)^.

Furthermore, LV remodeling after MS surgery is another critical issue, since knowledge of its pattern in terms of different preoperative echocardiographic parameters can offer a guide on the best timing for intervention. Previous studies^(8^,^9)^ evaluating MS patients submitted to percutaneous balloon mitral valvuloplasty identified a subgroup of patients with compromised left ventricular ejection fraction (LVEF) that showed no improvement compared to the control group, despite similar increases in mitral valve area (MVA). Although this is not a uniform finding, some authors have hypothesized that the increase in the end-diastolic volume could worsen an underlying dysfunction^(10)^.

The objectives of this study were to determine the pattern of LV remodeling, shortly after open mitral valve replacement for rheumatic MS, with in-hospital postoperative outcomes and the determinants of postoperative worsening of LVEF.

## METHODS

### Patients

From January 2008 to January 2015, 107 adult patients with pure rheumatic MS or mixed lesions with predominant MS were submitted to primary open mitral valve replacements, associated or not with tricuspid valve repairs and/or Cox-maze procedures. Exclusion criteria included more than moderate mitral regurgitation, aortic valve pathology, previous cardiac surgery, patients submitted to mitral valve repair, and those with incomplete follow-up.

Data were retrieved in part from the prospective cardiovascular surgery registry and in part from each electronic patient’s medical record. All echocardiographic analysis was performed in our institution by a team of qualified physicians following strict standardized protocols. Routine periodic interobserver variability is tested in our laboratory. Echocardiographic data were obtained from the echocardiography laboratory records. These data were approved by the Institutional Review Board (Ethics Committee approval number 2.644.241), which approved their use for research purposes with patient consent waived.

Operations were performed with normothermic cardiopulmonary bypass and hypothermic antegrade and retrograde blood cardioplegia for myocardial protection. Mitral valve replacement was performed with partial preservation of the subvalvular apparatus. The anterior leaflet was systematically resected. The posterior leaflet was preserved entirely or partially resected, in order avoid interference with the movement of the prosthesis leaflets and with LV filling. Secondary chordae were resected where extensive fibrotic tissue foreshortened the subvalvular apparatus, in addition to papillary muscle splitting to allow better ventricular filling. All calcified leaflet tissue was routinely removed. There were no patients in this series with mitral annular calcification in need of decalcification and reconstruction of the posterior atrioventricular groove.

### Echocardiographic Examination

All patients were submitted to a comprehensive two-dimensional and Doppler echocardiogram (HDI 5000, Philips ATL, Bothell, WA; HD 7, Philips, Koninklijke, N.V.; Vivid E9, GE) using 2/4 MHz transducer and second harmonic imaging, before and after any cardiothoracic surgical procedure in our unit. For the purpose of this research, the closest examinations performed before and after the surgery were reviewed. All postoperative examinations were completed prior to patient discharge.

LVEF was determined by the Simpson method. Left atrial volume (LAV) was calculated by the modified Simpson method and indexed to body surface areas. LAV was measured in the frame just before mitral valve opening, excluding the left atrial appendage and pulmonary veins.

MVA was measured by the planimetry method, using a parasternal short-axis view and pressure half-time (PHT), complying with the American Society of Echocardiography Guidelines^(11)^. Mean mitral valve diastolic gradients were measured by pulsed-wave Doppler. After valve replacement, patients were additionally evaluated for PHT and Doppler velocity index.

### Definitions of Study Groups and Outcomes

LV remodeling was studied through changes in LV dimensions, volumes, and function shortly after mitral valve replacement. For the purpose of this study, worsening of LV function was defined by a decrease in the LVEF of 10% or more between preoperative and postoperative echocardiographies. These criteria were based on the latest guidelines issued by the American Society of Echocardiography and the European Association of Cardiovascular Imaging^(12)^, which establishes a standard deviation of five absolute points for the mean normal values of LVEF, for both genders. That difference of > 10% was validated because it is greater than the interobserver accepted assessment error that may occur in echocardiography.

The outcomes assessed included intraoperative support (myocardial ischemic time, cardiopulmonary bypass time), as well as postoperative in-hospital mortality and morbidity rates (defined in accordance with the Society of Thoracic Surgeons National Database, available from: http://www.sts.org/registries-research-center/sts-national-database/adult-cardiac-surgery-database/data-collection).

### Data Analyses

All statistical analyses were performed using IBM Corp. Released 2016, IBM SPSS Statistics for Windows, Version 24.0, Armonk, NY: IBM Corp. Categorical variables are summarized as frequencies and percentages. The distribution patterns of variables were determined by the Kolmogorov-Smirnov test. Continuous variables are summarized as means and standard deviation when normally distributed or as medians with interquartile range when the data is skewed.

Categorical outcomes were compared using either χ^2^ or Fisher’s exact test (with less than five observations). Continuous outcomes were compared either by parametric tests (Student’s *t*-test) when variables had normal distribution, or by non-parametric tests (Mann-Whitney and Wilcoxon) when variable distribution was skewed. To compare temporal pattern of postoperative echocardiographic indices of dimensions and function across time, all repeated continuous values were analyzed longitudinally using repeated measures analyses of variance. Clinical and echocardiographic variables related to the development of postoperative worsening of LV function were determined by multivariable logistic regression. First, we determined factors associated with postoperative worsening of LV function using a univariable model. Variable selection, with a *P*-value criterion of 0.2 for retention of variables in the model, was followed by variable storage using automated forward selection and backward elimination. Receiver operating characteristic curves were obtained and the area under the curve was determined. A *P*-value of 0.05 was considered statistically significant.

## RESULTS

Overall, there was a marked predominance of women (N=93, 86.9%), and the mean age was 45±11 years (range from 22 to 71 years). More than half of the population had preoperative atrial fibrillation and they were in New York Heart Association (NYHA) functional class III or IV (Table 1). Mitral valve replacement was performed under mean cardiopulmonary bypass time of 93.7±29 minutes and mean cross-clamping time of 76±24.1 minutes, including associated procedures when applicable. Patients that presented with worsening EF after the procedure were those with lower preoperative body weight (*P*<0.01), that determined lower body mass index (*P*<0.01) and body surface area (*P*<0.01).

**Table 1:** Distribution of patients' preoperative characteristics.

	Total (N = 107)	Preserved EF (N = 89)	Worsening EF (N = 18)	*P*-value
Gender (male)	14 (13.1%)	13 (14.6%)	1 (5.6%)	0.54
Age^‡^	48 (38 - 53)	48 (39 - 53)	48 (33.7 - 59)	0.93
Weight (kg)^†^	61.6 ± 12.2	63.4 ± 12.1	52.9 ± 8.5	< 0.01
Height (m)^‡^	1.57 (1.54 - 1.62)	1.57 (1.54 - 1.63)	1.55 (1.50 - 1.61)	0.15
Body surface area^‡^	1.61 (1.52 - 1.72)	1.63 (1.54 - 1.74)	1.51 (1.43 - 1.57)	< 0.01
Body mass index^†^	24.5 ± 4.8	25.1 ± 4.9	21.6 ± 3.2	< 0.01
NYHA Class III or IV	58 (54.2%)	47 (55.3%)	11 (68.8%)	0.41
Hypertension	26 (24.3%)	23 (25.8%)	3 (16.7%)	0.55
Diabetes mellitus	6 (5.6%)	6 (6.7%)	0	0.59
Hyperlipidemia	6 (5.6%)	6 (6.7%)	0	0.59
Previous or current smoking	32 (29.9%)	27 (30.7%)	5 (27.8%)	> 0.999
Coronary artery disease	4 (3.7%)	3 (3.4%)	1 (5.6%)	0.53
Previous myocardial infarction	3 (2.8%)	2 (2.2%)	1 (5.6%)	0.43
Previous stroke	10 (9.3%)	8 (9%)	2 (11.1%)	0.67
Atrial fibrillation	54 (50.5%)	44 (49.4%)	10 (55.6%)	0.8

‡ Median (interquartile range)

† Mean ± standard deviation

EF=ejection fraction; NYHA=New York Heart Association

Intraoperative variables did not differ among study groups. Those include the type of prosthesis used (bioprosthesis in 59.1% of preserved EF group *vs.* 72.2% of worsening EF group; *P*=0.4), its sizing (28±1.9 *vs.* 27.3±1.4; *P*=0.2), presence of concomitant procedures - Maze procedure and/or tricuspid repair (43% *vs.* 44.4%; *P*=0.2) -, and duration of cardiopulmonary bypass time (94.2±28.3 minutes *vs.* 91.3±33.1 minutes; *P*=0.6) and aortic cross-clamping time (76.1±23.3 minutes *vs.* 75.3±28.2 minutes; *P*=0.8).

### Pattern of Ventricular Remodeling

Postoperatively (Table 2), there was an increase in LV dimensions and volumes, due to greater ventricular filling and relief of obstruction at mitral valve level, with decreased LAV and pulmonary artery pressures. Although the overall change in LVEF was significant (66.6% to 63.2%, *P*<0.01), its means were in the normal range. Worsening of LVEF > 10% occurred in 18 patients (16.8%) and only six patients (5.6%) presented EF < 50%.

**Table 2:** Variation of echocardiographic data among preoperative and postoperative periods.

	Preserved EF (N = 89)			Worsening EF (N = 18)			Total (N = 107)		
	Preoperative^‡^	Postoperative^‡^	*P*-value	Preoperative^‡^	Postoperative^‡^	*P*-value	Preoperative^‡^	Postoperative^‡^	*P*-value
Indexed LVEDD (mm/m^2^)	27.3 (25.2 - 29.4)	27.9 (26.2 - 29.8)	< 0.01	28.2 (24.6 - 31.2)	29.6 (26.3 - 33.5)	0.10	27.5 (24.8 - 29.5)	27.9 (26.2 - 30.32)	< 0.01
Indexed LVESD (mm/m^2^)	17.6 (16.1 - 19.0)	18.1 (16.0 - 19.3)	0.03	16.8 (14.2 - 18.7)	19.4 (18.4 - 24.5)	< 0.01	17.4 (15.8 - 18.9)	18.3 (16.2 - 19.6)	< 0.01
Indexed LVEDV (ml/m^2^)	57.3 (45.8 - 66.8)	58.7 (50.8 - 67.9)	< 0.01	52.2 (39.8 - 62.8)	62.8 (49.4 - 75.0)	0.05	57.1 (45.1 - 66.2)	58.7 (50.5 - 68.5)	< 0.01
Indexed LVESV (ml/m^2^)	20.3 (15.1 - 22.9)	20.7 (16.2 - 24.4)	0.05	16.3 (10.2 - 20.3)	23.5 (19.6 - 39.2)	< 0.01	19.4 (14.9 - 22.8)	20.9 (16.8 - 26.8)	< 0.01
LVEF (%)	64.4 (61.6 - 68.5)	65.3 (61.0 - 69.1)	0.58	71.7 (65.5 - 79.0)	58.4 (33.9 - 62.4)	< 0.01	65.8 (62.1 - 68.7)	64 (59.8 - 68.5)	0.02
Indexed LA volume (ml/m^2^)	61.3 (50.5 - 73.7)	45.6 (40.0 - 55.6)	< 0.01	63.7 (48.8 - 81.8)	53.3 (39.3 - 78.1)	< 0.01	62.5 (50.5 - 74.2)	46 (40 - 56.4)	< 0.01
Mean mitral gradient (mmHg)	12.0 (8.5 - 17.0)	6.2 (4.1 - 8.1)	< 0.01	15.5 (11.7 - 19.2)	6.0 (3.8 - 8.2)	< 0.01	13.0 (9 - 18)	6.0 (4.0 - 8.0)	< 0.01
PASP (mmHg)	50.0 (40.0 - 62.0)	38.0 (35.0 - 47.5)	< 0.01	52.0 (43.0 - 83.0)	48.0 (34.5 - 55.5)	0.08	51.5 (40 - 62)	39.0 (35.0 - 49.5)	< 0.01

‡ Median (interquartile range)

EF=ejection fraction; LA=left atrial; LVEDD=left ventricular end-diastolic diameter; LVEDV=left ventricular end-diastolic volume; LVEF=left ventricular ejection fraction; LVESD=left ventricular end-systolic diameter; LVESV=left ventricular end-systolic volume; PASP=pulmonary artery systolic pressure

**Fig. 1:**
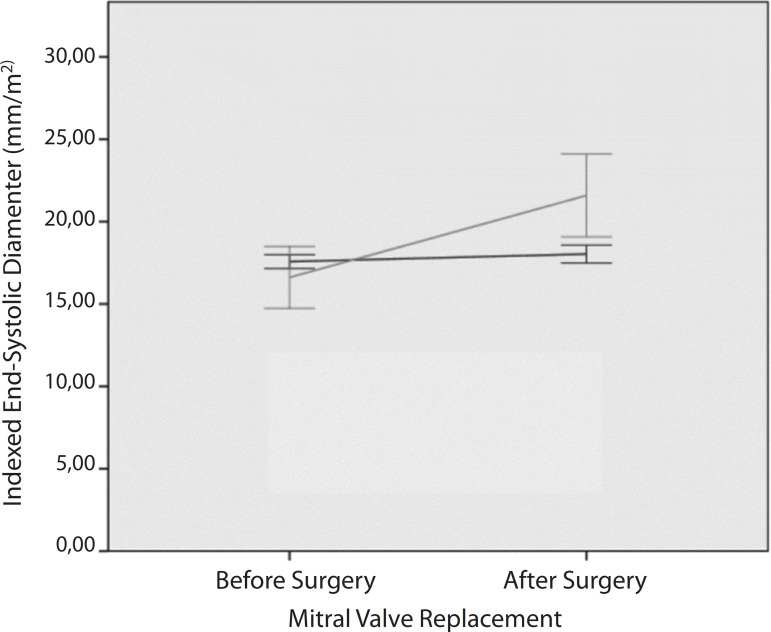
Indexed left ventricular end-systolic diameter pattern of remodeling after mitral valve replacement according to the study group (dark line=preserved ejection fraction [EF] group; light line=worsening EF group).

**Fig. 2:**
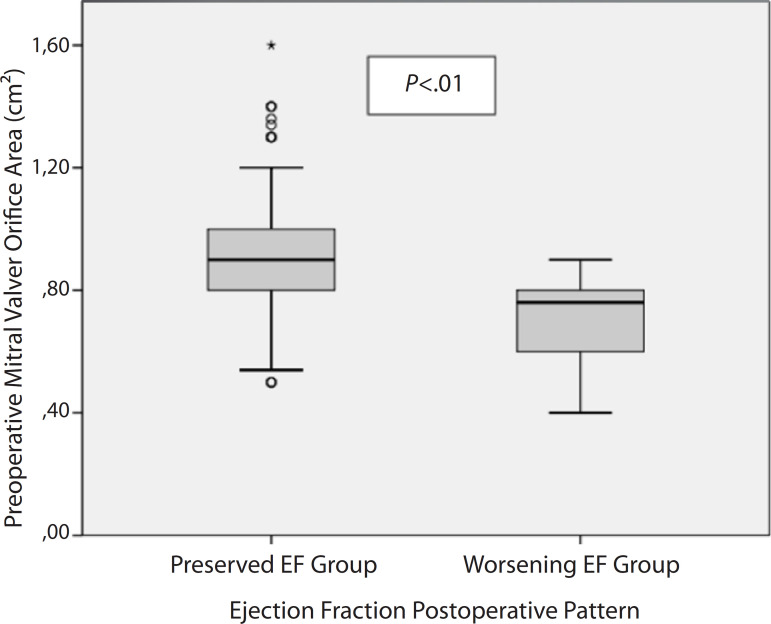
Mitral valve orifice area before surgery according to the study group. EF=ejection fraction.

When the groups were compared (Table 3), LV end-diastolic diameters (*F*=0.04; *P*=0.83) and volumes (*F*=0.75; *P*=0.38) increased similarly. The increase in the end-systolic volume (*F*=34; *P*<0.01), as shown in Figure 1, and diameter (*F*=43.1; *P*<0.01) was far greater in the group that presented worsening of EF. Moreover, the mitral valve orifice area (Figure 2) was smaller in the group with deteriorated EF, indicating a more severe MS. Obstruction relief postoperatively was greater in the same group (*F*=4.5; *P*=0.03). Both groups presented similar decreases in LAV (*F*=23.9; *P*=0.2) and proportional improvements in pulmonary artery systolic pressures (*F*=0.38; *P*=0.53).

**Table 3:** Distribution of echocardiographic data among study groups.

	Preoperative				Postoperative			
	Total (N = 107)	Preserved EF (N = 89)	Worsening EF (N = 18)	*P*-value	Total (N = 107)	Preserved EF (N = 89)	Worsening EF (N = 18)	*P*-value
Indexed LVEDD (mm/m^2^)^‡^	27.5 (24.8 - 29.5)	27.3 (25.2 - 29.4)	28.2 (24.6 - 31.2)	0.48	27.9 (26.2 - 30.32)	27.9 (26.2 - 29.8)	29.6 (26.3 - 33.5)	0.05
Indexed LVESD (mm/m^2^)^‡^	17.4 (15.8 - 18.9)	17.6 (16.1 - 19.0)	16.8 (14.2 - 18.7)	0.18	18.3 (16.2 - 19.6)	18.1 (16.0 - 19.3)	19.4 (18.4 - 24.5)	< 0.01
Indexed LVEDV (ml/m^2^)^‡^	57.1 (45.1 - 66.2)	57.3 (45.8 - 66.8)	52.2 (39.8 - 62.8)	0.40	58.7 (50.5 - 68.5)	58.7 (50.8 - 67.9)	62.8 (49.4 - 75.0)	0.5
Indexed LVESV (ml/m^2^)^‡^	19.4 (14.9 - 22.8)	20.3 (15.1 - 22.9)	16.3 (10.2 - 20.3)	0.01	20.9 (16.8 - 26.8)	20.7 (16.2 - 24.4)	23.5 (19.6 - 39.2)	0.03
LVEF (%)^‡^	65.8 (62.1 - 68.7)	64.4 (61.6 - 68.5)	71.7 (65.5 - 79.0)	< 0.01	64.04 (59.8 - 68.5)	65.3 (61.0 - 69.1)	58.4 (33.9 - 62.4)	< 0.01
Indexed LA volume (ml/m^2^)^‡^	62.5 (50.5 - 74.2)	61.3 (50.5 - 73.7)	63.7 (48.8 - 81.8)	0.73	46.0 (40.0 - 56.4)	45.6 (40.0 - 55.6)	53.3 (39.3 - 78.1)	0.17
Mean mitral gradient (mmHg)^‡^	13.0 (9.0 - 18.0)	12.0 (8.5 - 17.0)	15.5 (11.7 - 19.2)	0.02	6.0 (4.0 - 8.0)	6.2 (4.1 - 8.1)	6.0 (3.8 - 8.2)	0.65
MVA (cm^2^)^‡^	0.80 (0.70 - 0.99)	0.9 (0.8 - 1.0)	0.76 (0.60 - 0.80)	< 0.01	-	-	-	-
Indexed MVA (cm^2^/m^2^)^‡^	0.52 (0.44 - 0.61)	0.52 (0.45 - 0.62)	0.49 (0.40 - 0.54)	0.08	-	-	-	-
Wilkins-Block^‡^	10.0 (9.0 - 11.0)	10 (9.0 - 11.0)	11.0 (10.0 - 12.0)	0.05	-	-	-	-
PHT (ms)^‡^	-	-	-	-	72.0 (52.0 - 100.0)	71.0 (51.7 - 95.0)	75 (60.5 - 245.5)	0.36
VTIPrMV/VTILVOT^†^	-	-	-	-	1.9 ± 0.5	2.0 ± 0.5	1.9 ± 0.5	0.74
PASP (mmHg)^‡^	51.5 (40.0 - 62.0)	50.0 (40.0 - 62.0)	52.0 (43.0 - 83.0)	0.27	39.0 (35.0 - 49.5)	38.0 (35.0 - 47.5)	48.0 (34.5 - 55.5)	0.06
Moderate MR	37 (34.6%)	30 (33.7%)	7 (38.9%)	0.79	-	-	-	-
Moderate or severe TR	26 (24.3%)	19 (21.3%)	7 (38.9%)	0.14	-	-	-	-

‡ Median (interquartile range)

† Mean (95% confidence interval)

EF=ejection fraction; LA=left atrial; LVEDD=left ventricular end-diastolic diameter; LVEDV=left ventricular end-diastolic volume; LVEF=left ventricular ejection fraction; LVESD=left ventricular end-systolic diameter; LVESV=left ventricular end-systolic volume; MR=mitral regurgitation; MVA=mitral valve area; PASP=pulmonary artery systolic pressure; PHT=pressure half-time; TR=tricuspid regurgitation; VTILVOT=velocity-time integral for left ventricle outflow tract; VTIPrMV=velocity-time integral for mitral valve prosthesis

### Determinants of Worsening of Left Ventricular Ejection Fraction

Body weight (*P*=0.005; odds ratio [OR]=0.89) and MVA (*P*=0.02; OR=0.02) were identified as independent predictors of worsening of LVEF. Moderate mitral regurgitation was not associated with worsening of postoperative EF.

A receiver operating characteristic curve was then obtained for the MVA by planimetry. Regarding the non-indexed MVA, the optimal cutoff for prognostic value was determined by the model as 0.8 (sensitivity=88.2%, specificity=54.4%), with an overall area under the curve of 0.752 (95% confidence interval 0.645 - 0.859; *P*<0.01).

### Surgical Outcomes

Postoperative in-hospital morbidity and mortality rates are depicted in Table 4. Patients in the group with worsening LVEF presented higher mortality rates, more prolonged mechanical ventilation and nosocomial pneumonia, and prolonged hospital and intensive care unit lengths of stay. Postoperatively, this group developed more frequently hemodynamic instability with elevated filling pressures. Causes of death of this group were cardiogenic shock in all three patients.

**Table 4:** In-hospital surgical outcomes of patients submitted to mitral valve replacement according to study groups.

	Total (N = 107)	Preserved EF (N = 89)	Worsening EF (N = 18)	OR (95% CI)	*P*-value
In-hospital mortality	4 (3.7%)	1 (1.1 %)	3 (16.7%)	17.6 (1.7 - 181)	0.01
Stroke	0	0	0	-	-
Myocardial infarction	0	0	0	-	-
Acute renal failure	7 (6.5%)	4 (4.5%)	3 (16.7%)	4.2 (.8 - 20.7)	0.08
New-onset atrial fibrillation	19 (17.7%)	18 (20.5%)	1 (5.6%)	.23 (.02 - 1.83)	0.16
Prolonged mechanical ventilation	5 (4.6%)	1 (1.1%)	4 (22.2%)	24.8 (2.6 - 238)	<0.01
Nosocomial pneumonia	5 (4.6%)	2 (2.3%)	3 (16.7%)	8.6 (1.3 - 55.8)	0.03
ICU length of stay (days)^‡^	4 (2 - 5)	3 (2 - 3)	6 (3 - 8)	-	0.03
Hospital length of stay (days)^‡^	13 (9 - 17)	12 (9 - 17)	15 (11 - 18)	-	0.04

‡ Median (interquartile range)

CI=confidence interval; EF=ejection fraction; ICU=intensive care unit; OR=odds ratio

## DISCUSSION

This study shows that there are different patterns of LV remodeling postoperatively among patients with rheumatic MS submitted to open mitral valve replacement. Worsening LVEF > 10% is uncommon, but when present may lead to higher morbidity and mortality rates. Predictors of worsening LVEF relate to a specific subset of patients with lower body weight and smaller mitral valve orifice areas.

Patient profiles showed a predominance of women in their forties presenting as very symptomatic (nearly half in NYHA functional class > III) and about half with preoperative atrial fibrillation. Patients submitted to mitral valve repair were excluded since this could be a confounder when analyzing LV remodeling patterns.

The pattern of ventricular remodeling in patients presenting with worsening of LVEF was associated with greater ventricular dilatation. Moreover, they also had smaller preoperative mitral valve orifice areas, indicating more severe MS at presentation. This finding might be related to poor adaptation by the left ventricle to a subtle increase in end-diastolic volumes. Diastolic dysfunction is usually the explanation for that ^(13)^, but underlying systolic dysfunction could be unmasked after surgery. LVEF has limitations for defining the degree of systolic ventricular dysfunction^(14)^. Echocardiographic assessment with speckle tracking echocardiography has demonstrated that longitudinal and circumferential strain rates are globally diminished in MS patients^(15)^, even with preserved EF.

Lower body weight and smaller preoperative mitral valve orifice areas were related to worsening of LVEF postoperatively. The association between severity of MS and ventricular dysfunction is conflictive. Previous studies have shown a linear relationship between MVA in severe MS patients and systolic and diastolic myocardial velocities determined by Doppler tissue imaging^(16)^,^(17)^. Moreover, a positive correlation was found between MVA and impaired longitudinal strain rates^(18)^. However, these results are not consistent in patients with mild to moderate MS^(15^,^19)^.

The 2014 American Heart Association/American College of Cardiology Guidelines^(20)^ on valvular heart disease recommend that the presence of symptoms with evidence of severe MS (MVA < 1.5 cm^2^, stage D) is necessary as an indication for surgery. It is also considered reasonable as an indication for percutaneous balloon valvuloplasty (rather than surgery) a Recommendation Class IIa in asymptomatic patients with very severe MS (MVA < 1.0 cm^2^, stage C) and favorable morphology, in the absence of contraindications. This recommendation stresses the incremental risk of very severe MS (MVA < 1.0 cm^2^) over severe stenosis (MVA < 1.5 cm^2^), even in stage C patients. The 2017 European Society of Cardiology/European Association for Cardio-Thoracic Surgery Guidelines^(21)^ on valvular disease recommend as an indication that intervention in asymptomatic patients be performed only percutaneously, with high thromboembolic risk and/or high risk of hemodynamic decompensation, at the same recommendation level as the American guidelines. The Brazilian Society of Cardiology Guidelines^(22)^ recommend intervention as Class IIa in asymptomatic patients, only in the presence of pulmonary hypertension and/or new-onset atrial fibrillation, if not suitable for percutaneous balloon valvuloplasty. Our data supports this indication for surgery based on symptoms alone or with complicating factors (new-onset atrial fibrillation, pulmonary hypertension, or desire for pregnancy) that may be misleading, particularly when MVA < 1.0 cm^2^.

Our data showed that worsening of LVEF > 10% is associated with poorer in-hospital outcomes. Similarly, proper assessment of LV systolic and diastolic function is important for determining surgical risk and prognosis. Early intervention seems reasonable for this patient subset. In our study, MVA of 0.80 cm^2^ presented good sensitivity (88.2%) and low specificity (54.4%), raising concerns about very severe MS and postoperative subclinical ventricular dysfunction.

### Limitations

This is a retrospective patient cohort study, with its inherent limitations. The low number of events curtailed a more appropriate analysis of outcomes with multivariable logistic regression, particularly those that achieve worsening LVEF (N=19). However, a fair number of rheumatic MS patients were included. The importance of this study lies in the recognition that there is a subset of patients who develop clinically relevant LV adverse remodeling. Long-term clinical and echocardiographic data would be extremely important for understanding whether ventricular remodeling pattern persists over time, together with its impact on survival.

## CONCLUSION

LV remodeling patterns differed among patients with predominant rheumatic MS undergoing open mitral valve replacement. Lower preoperative body weight and MVA were independent determinants of deteriorating EF with increased end-systolic volumes, indicating that this specific problem may occur in anthropometric smaller patients with more extensive rheumatic disease.

**Table:** 

Authors' roles & responsibilities	
MVSF	Substantial contributions to the conception or design of the work; or the acquisition, analysis, or interpretation of data for the work; drafting the work or revising it critically for important intellectual content; agreement to be accountable for all aspects of the work in ensuring that questions related to the accuracy or integrity of any part of the work are appropriately investigated and resolved; final approval of the version to be published
CRC	Substantial contributions to the conception or design of the work; or the acquisition, analysis, or interpretation of data for the work; drafting the work or revising it critically for important intellectual content; agreement to be accountable for all aspects of the work in ensuring that questions related to the accuracy or integrity of any part of the work are appropriately investigated and resolved; final approval of the version to be published
GSO	Substantial contributions to the conception or design of the work; or the acquisition, analysis, or interpretation of data for the work; drafting the work or revising it critically for important intellectual content; agreement to be accountable for all aspects of the work in ensuring that questions related to the accuracy or integrity of any part of the work are appropriately investigated and resolved; final approval of the version to be published
MEO	Substantial contributions to the conception or design of the work; or the acquisition, analysis, or interpretation of data for the work; drafting the work or revising it critically for important intellectual content; agreement to be accountable for all aspects of the work in ensuring that questions related to the accuracy or integrity of any part of the work are appropriately investigated and resolved; final approval of the version to be published
FAA	Substantial contributions to the conception or design of the work; or the acquisition, analysis, or interpretation of data for the work; drafting the work or revising it critically for important intellectual content; agreement to be accountable for all aspects of the work in ensuring that questions related to the accuracy or integrity of any part of the work are appropriately investigated and resolved; final approval of the version to be published
